# Tumor Targeting Effect of Triphenylphosphonium Cations and Folic Acid Coated with Zr-89-Labeled Silica Nanoparticles

**DOI:** 10.3390/molecules25122922

**Published:** 2020-06-25

**Authors:** Gun Gyun Kim, Jun Young Lee, Pyeong Seok Choi, Sang Wook Kim, Jeong Hoon Park

**Affiliations:** 1 Department of Advanced Materials Chemistry, Dongguk University, Gyeongju 38066, Korea; gungyun88@dongguk.ac.kr; 2 Radiation Utilization and Facilities Management Division, Korea Atomic Energy Research Institute, Jeongeup 56212, Korea; ljy01@kaeri.re.kr (J.Y.L.); parkjh@kaeri.re.kr (P.S.C.)

**Keywords:** Zr-89, PET imaging, triphenylphosphonium cation, folic acid, silica nanoparticles, surface modification

## Abstract

In this study, we investigated the tumor targeting effect in cancer cells using triphenylphosphonium (TPP) cations, which are accumulated by differences in membrane potential, and folic acid (FA), which is selectively bound to overexpressed receptors on various cancer cells. We used Food and Drug Administration (FDA)-approved silica nanoparticles (SNPs) as drug carriers, and SNPs conjugated with TPP and FA (STFs) samples were prepared by introducing different amounts of TPP and FA onto the nanoparticle surfaces. STF-1, 2, 3, 4 and 5 are named according to the combination ratio of TPP and FA on the particle surface. To confirm the tumor targeting effect, ^89^Zr (t_1/2_ = 3.3 days) was coordinated directly to the silanol group of SNP surfaces without chelators. It was shown that the radiochemical yield was 69% and radiochemical purity was >99%. In the cellular uptake evaluation, SNPs with the most TPP (SFT-5) and FA (SFT-1) attached indicated similar uptake tendencies for mouse colon cancer cells (CT-26). However, the results of the cell internalization assay and measurement of positron emission tomography (PET) images showed that SFT-5 had more affinity for the CT-26 tumor than other samples the TPP ratio of which was lower. Consequently, we confirmed that TPP ligands affect target cancer cells more than FA, which means that cell membrane potential is significantly effective for tumor targeting.

## 1. Introduction

Triphenylphosphonium (TPP) cations are lipophilic cations that can target cancer cells through the membrane potential of cancer cells [1,2]. Liposoluble TPP can easily pass through lipid bilayer membranes, and its cationic molecules exhibit several-hundred-fold higher uptakes in mitochondria with negative membrane potentials (−150 to −170 mV) than extracellular fluids [3,4,5]. Mitochondria in cancer cells are known to have a membrane potential difference of about 60 mV from normal cells due to abnormal metabolic behavior, and 10 times more TPP accumulates in the mitochondria of cancer cells due to this difference [6,7,8]. Folic acid (FA) can be internalized by the folate receptor (FR) expressed on the cell membrane in the body [9,10,11]. Among several FRs, FR-α is overexpressed in about 40% of human cancers, and FR-β is expressed on the surface of activated macrophages and hematopoietic stem cells [12,13]. FA and FA complexes show high affinity (K_d_ ~ 10^−9^M) for FR-α and FR-β and are endogenous to FR-expressing cells because of their receptor-mediated endocytosis. Studies on the targeted treatment and diagnosis of various cancers using these characteristics of FA have been reported [14,15].

The research and development of drug delivery systems (DDSs) is very important in the life sciences. The first commercial DDS was approved in 1990 by the US Food and Drug Administration (FDA) to contain amphotericin B, an antimicrobial agent, and more than 10 DDSs were marketed thereafter [16]. Effective DDSs should increase the bioavailability of the drug and minimize side effects [17]. In particular, the system should target the disease site with an effective concentration and lower the damage to normal tissue by regulating the release rate of the drug in the body and maintaining the concentration [18,19]. Since nanocarrier-based therapies can overcome the limitations of existing therapies, anticancer agents targeting specific cancers have been actively studied. The main mechanism for tumor targets in nanocarriers is the enhanced permeability and preservation (EPR) effect of accumulating drug carriers through incomplete capillaries formed around the tumor [20,21]. Among these nanocarriers, silica nanoparticles (SNPs) have a hydrophobic surface, can circulate in the blood for a long time, and can introduce various chemical functional groups to the surface simply [22,23,24,25]. They are also widely used in the field of drug delivery due to their excellent biocompatibility and the possibility of their mass production with a uniform size and shape. In 2011, the Investigational New Drug Application was first approved by the FDA for clinical trials [26,27].

TPP has an effective cellular uptake mechanism caused by cell membrane potential differences, enabling tumor targeting studies to be actively carried out [28]. FA is widely used in receptor-mediated cellular uptake research as a ligand that can target FRs that are overexpressed in various cancer cells. The tumor targeting effects of the two types of compounds are well known, but no studies have directly compared them. Therefore, it is necessary to combine the two compounds for tumor targeting with different mechanisms to nanoparticles, compare drug delivery effects through various biological evaluations, and confirm the possibility of developing DDS.

**Scheme 1:**
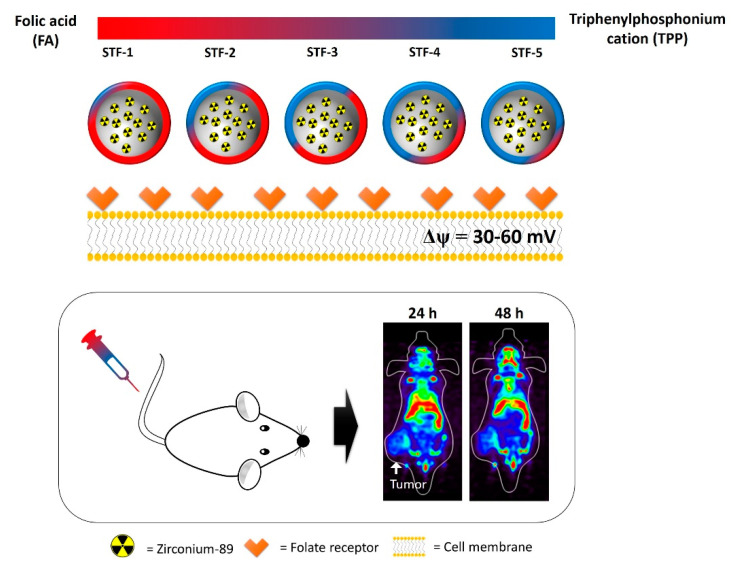
Total scheme of biological evaluation for ^89^Zr-labeled STF-1 to 5.

In this study, TPP and folic acid were conjugated at different ratios on the surfaces of SNPs. Zirconium-89 (^89^Zr, t_1/2_ = 3.3 days) was introduced without a chelator on SNP, and the drug delivery efficiency of SNPs varied according to the ratio of TPP to FA (Scheme 1), which was analyzed over time using positron emission tomography (PET) [29,30,31].

## 2. Results

### 2.1. Synthesis of TPP and FA Conjugated SNPs

**Figure 1:**
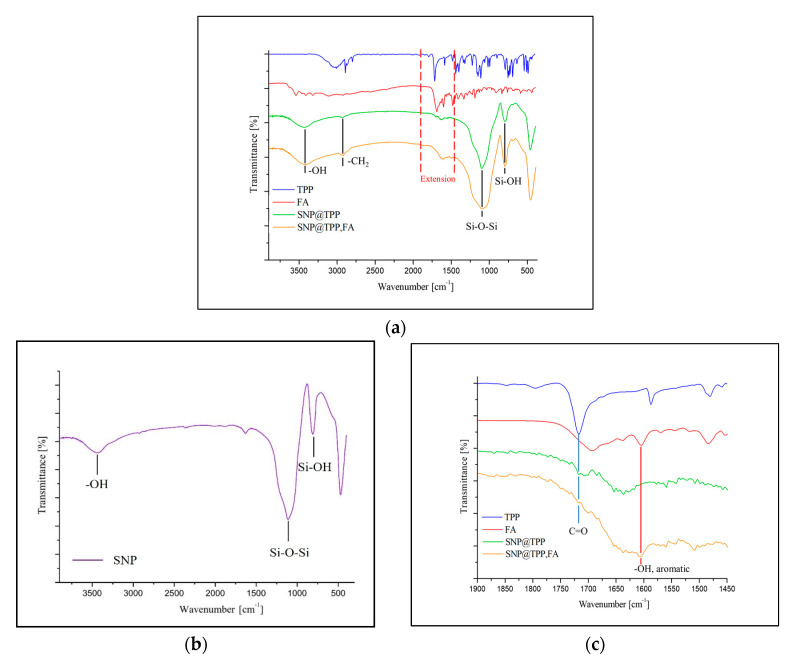
(**a**) FT-IR spectrum of triphenylphosphonium (TPP)- and folic acid (FA)-modified silica nanoparticles (SNPs); (**b**) silica nanoparticle; (**c**) extension spectrum (**a**) at 1450 to 1900 cm^−1^.

SNPs were synthesized by the method of Stöber and analyzed by FT-IR and an SEM. In the FT-IR measurement, the hydroxy group on the surface of the SNPs was confirmed as about 3450 cm^−1^, and the peaks of Si-O-Si and Si-OH were confirmed as about 1119 and 808 cm^−1^ (Figure 1a,b). In the spectrum of 1720 cm^−1^ in Figure 1c, the peak of the carbon and oxygen double bond of TPP can be confirmed on the surface of the SNP, and a hydroxyl group bound to the aromatic ring of FA can be seen at around 1607 cm^−1^.

**Figure 2:**
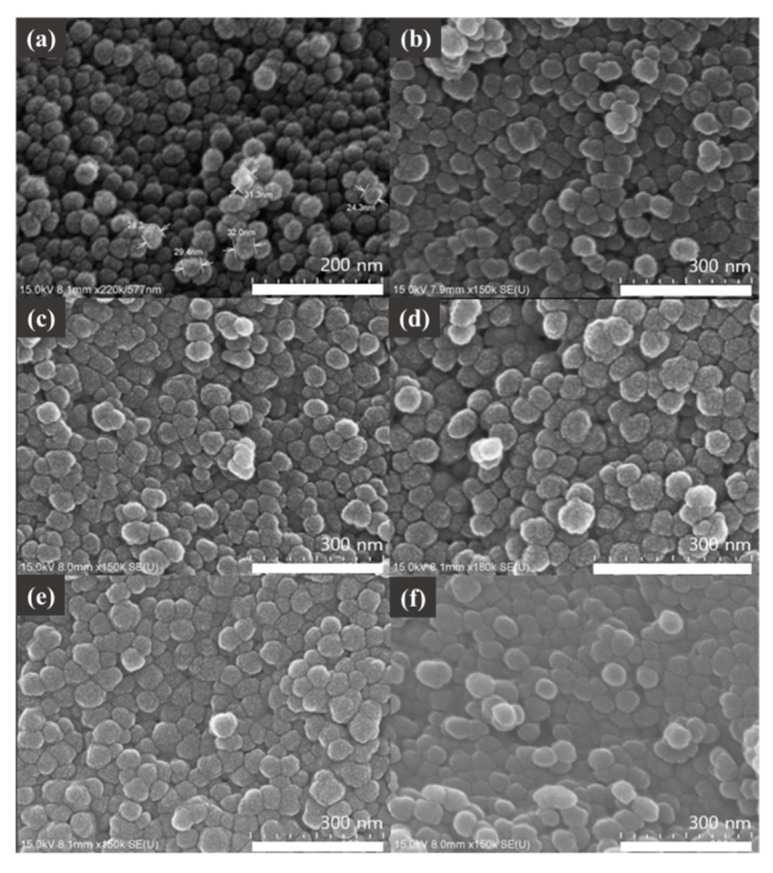
Scanning electron microscope (SEM) images of (**a**) SNPs, (**b**) STF-1, (**c**) STF-2, (**d**) STF-3, (**e**) STF-4, and (**f**) STF-5.

The spherical shape of the SNPs synthesized by the SEM was found to be uniform, and all of them were about 30 nm in diameter, although the number of compounds bonded to the surface of the nanoparticles differed, as shown in A to F in Figure 2.

**Figure 3:**
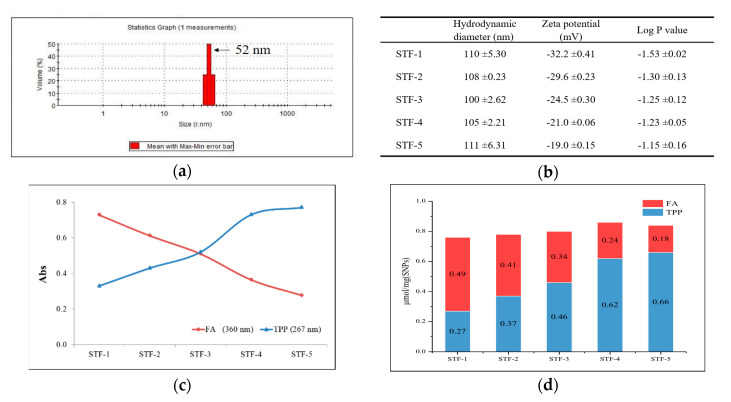
Dynamic light scattering (DLS) analysis and spectroscopic analysis of nanoparticles. (**a**) Hydrodynamic diameter of SNPs in distilled water. (**b**) Dynamic light scattering studies and Log P value of STF-1 to 5. (**c**) UV absorbance of 0.1 mg/mL STFs in distilled water. (**d**) The amount of FA and TPP on SNP surfaces (μmol/mg).

The hydrodynamic diameters of SNPs were measured using a zeta-sizer (Nano ZS90, Malvern) and were found to be around 50 nm (Figure 3a). The hydrodynamic diameters of SNPs conjugated with TPP and FA are listed in Figure 3b. The compounds were named STF-1 to 5 according to the binding amount. TPP and FA were introduced on the surfaces of SNPs, and the presence of TPP and FA on the particle surface could be confirmed by UV-Vis measurement. The binding amount of each compound was calculated using a calibration curve prepared in advance, and the result is shown in Figure 3c,d. Dynamic light scattering studies and Log P values of each sample are shown in Figure 3b.

### 2.2. Zr-89 Radiolabeling 

After the Zr-89 labeling reaction, the labeling yield and radiochemical purity were evaluated using a Radio-TLC reader with DTPA as the developing solvent, and the results are shown in Table 1. The labeling yield of ^89^Zr for STF-1 to 5 was 69.4% ± 1.43%, and the radiochemical purity after centrifugation was 99.5% ± 0.46%.

### 2.3. In-Vitro Studies: Cytotoxicity and Stability Test

**Figure 4:**
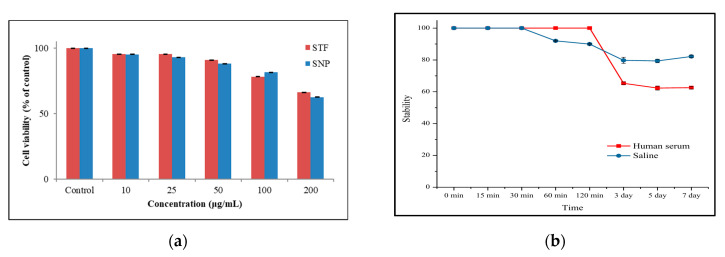
(**a**) The cytotoxicity study using STF and SNP was conducted on CT-26 for 24 h and (**b**) In-vitro stability evaluation of ^89^Zr-STF: Stability in human serum and saline was monitored by radio TLC. The ^89^Zr -STF was incubated with 1.0 mL of human serum and saline at 37 °C for 7 days and then analyzed.

In the in-vitro studies, the cytotoxicity of the sample was evaluated using an MTT assay, and TPP- and FA-modified SNPs were compared with unmodified SNPs. As a result, it was confirmed that the sample modified on the surface was similar to that of untreated SNPs and showed cell viability of more than 66% at the highest concentration (Figure 4a). Stability in human serum and saline was evaluated, and a stability of 90% or more was maintained for 2 h. After 7 days, the stability in saline was still 80% or more, but the stability in human serum was 62% (Figure 4b).

### 2.4. Cellular Uptake and Cell Internalization Assay

**Figure 5:**
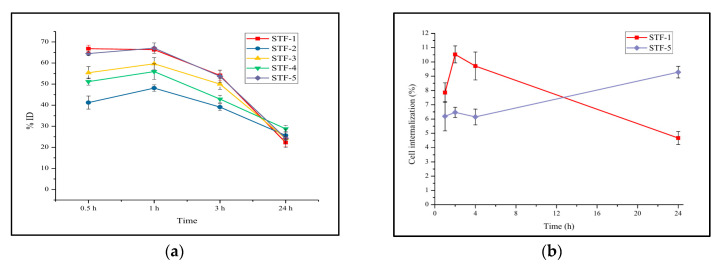
Results of (**a**) cellular uptake for STF-1~5 and (**b**) cell internalization for STF 1 and 5.

Cellular uptake was evaluated using CT-26, the mouse colon cancer cell line, and evaluated over 24 h. The evaluation subjects were STF-1 to STF-5. The highest cell uptake was observed at 1 h after sample addition and gradually decreased over 24 h. The reason why the uptake of all samples decreases after 1 h is thought to be the release of free ^89^Zr due to the labelling stability of ^89^Zr. This is the result of labeling without chelators to prevent the change in the properties of the nanoparticle surface, and it is thought that labeling stability can be improved by introducing chelators. Among the samples, STF-1 and 5 showed a relatively high uptake. It was confirmed that the effects of folic-acid-receptor-mediated cell uptake and cell uptake by membrane potential differences were similar, and the difference in effectiveness between the two samples was investigated through an in-vivo behavior study (Figure 5a). In the additional evaluation of STF-1 and STF-5 internalization, STF-1 showed the maximum cell internalization (10.5%) over a short time and then gradually decreased, whereas STF-5 continuously increased the internalization (9.3%) over 24 h (Figure 5b).

### 2.5. In-Vivo Study: PET Imaging

**Figure 6:**
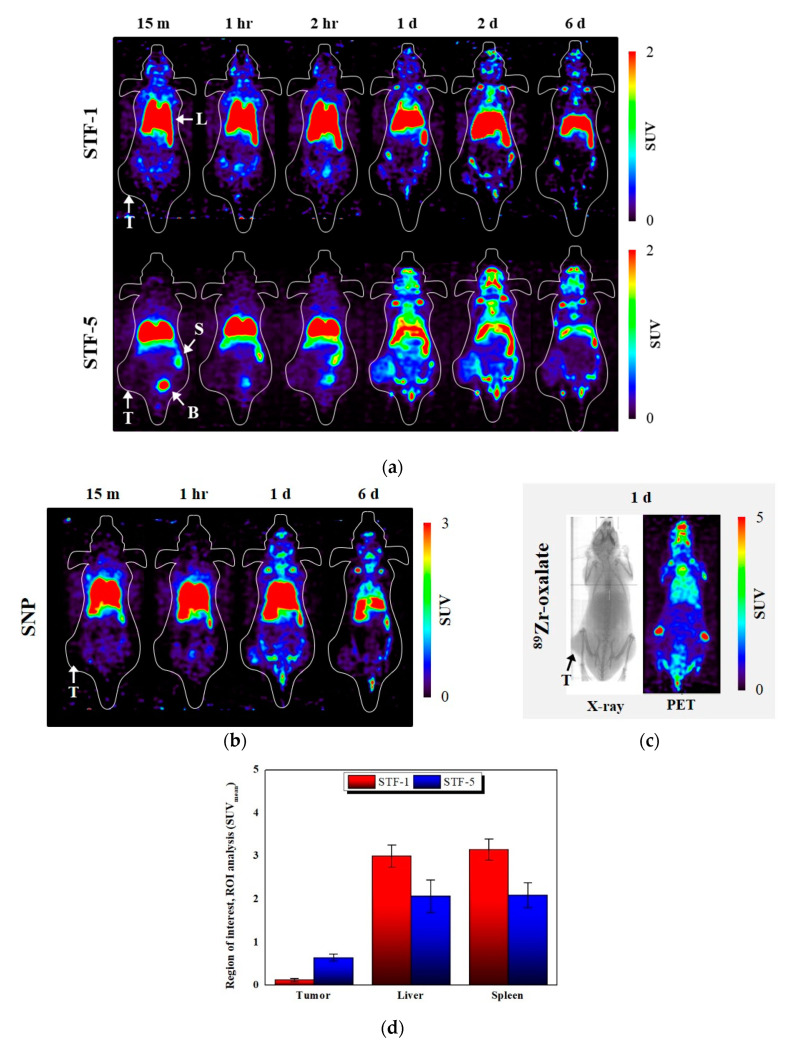
Positron emission tomography (PET) study results of CT-26-bearing 5-week-old female Balb/c mice. CT-26 xenograft mice were scanned for a 5-min static image at time points after intravenous injection of (**a**) ^89^Zr-STF-1 and ^89^Zr-STF-5; (**b**) ^89^Zr-SNP; (**c**) ^89^Zr-oxalate; T: tumor, L: liver, S: spleen, B: bladder; (**d**) ROI analysis of STF-1 and STF-5 one day after injection.

In the PET study, Balb/c mice modeled using the mouse colon cancer cell line CT-26 for cell uptake were used, and STF-1 and STF-5 were evaluated. STF-1 and STF-5 accumulated in the liver and spleen after the tail vein injection. This type of accumulation was a typical uptake for the reticuloendothelial system (RES) and was maintained for 6 days. After 1 day, STF-5 was accumulated at the cancer site. In addition, at the beginning of the evaluation, clearance through the bladder was observed in STF-5, and both samples showed a high accumulation in bone depending on the time of measurement. Accumulation in bone is characteristic of ^89^Zr, which has an affinity for bone and is also related to the stability of the ^89^Zr-labeled nanoparticles in the body (Figure 6a). In Figure 6d, the results of the region of interest (ROI) analysis after one day of injection showed that STF-5 identified a higher radioactivity intensity on the tumor compared to STF-1, while this was lower for the liver and spleen. We would like to compare the surface with the PET image of the ^89^Zr-labeled SNP without TPP and FA combined. The ^89^Zr-labeled SNP showed a RES accumulation, and a day later it had an accumulation in the tumor, which is an accumulation due to the EPR effect (Figure 6b). Figure 6c shows the in-vivo behavior of free ^89^Zr by injection of ^89^Zr-oxalate. We could see that most ^89^Zr accumulates in the bones, and this result means that the accumulation we present for tumors is not caused by free ^89^Zr but by the ^89^Zr-labeled nanoparticles.

## 3. Discussion

The peak of the hydroxyl group (3450 cm^−1^) was confirmed by the FT-IR spectrum of the synthesized SNPs. It is considered that the hydroxy peak is a peak of the hydroxyl group present on the surface of the SNP after completion of the condensation reaction of the hydrolyzed silanol groups of the TEOS used as the Si source. The intrinsic strong stretching vibration peak (Si-O-Si) of the SNPs can be confirmed as 1119 cm^−1^, and the peak of Si-OH at 808 cm^−1^ was also confirmed. TPP and FA bound to the SNP surface can be checked for synthesis through C=O and -OH bond peaks of 1720 and 1607 cm^−1^. An SEM was used to determine the particle morphology of the SNPs. The synthesized SNPs were particles with a uniform diameter of 30 nm, and the shape was confirmed to be spherical. Spherical nanoparticles were also observed in the SEM image after the binding of TPP to FA on the surface of the nanoparticles. The diameter of the spherical nanoparticles was about 60 nm, which was larger than that of the non-surface-modified SNPs. There was no morphological difference between the five surface-modified samples. In addition, the surface-modified SNPs showed an average size of 50 nm in the DLS measurement, and the surface-modified samples were found to have an average size of 106 nm.

UV-Vis spectroscopy was used to determine the amount of TPP and FA bound to the surfaces of the SNPs. The maximum absorption peak of TPP was confirmed as 267 nm, and the absorption wavelength of folic acid was confirmed as 360 nm to avoid overlap with the absorption peak of TPP. The absorbance of each sample was as shown in Figure 3c, and the amount of TPP and FA bound per mg of sample could be calculated using a pre-prepared calibration curve. The amount of TPP bound from STF-1 to STF-5 was increased, while the amount of FA was decreased. The zeta potential of the surface-modified samples was measured. All five samples had a negative charge, and the samples with a high binding amount of TPP had a low negative charge because TPP offset the negative charge on the surface. We were able to identify the change in the surface charge of the nanoparticles and the change in Log P values due to the combination ratio of TPP and FA, and we would like to identify the in-vivo behavior of nanoparticles that can be derived when the two compounds are introduced together.

In the comparison of the cell survival rates of the SNPs and the surface-modified samples, the difference between the two samples was small, and the survival rate was confirmed to be 60% or more, even at a concentration of 200 μg/mL. These results indicate that the nanoparticles modified with TPP and FA have a similar cytotoxicity to that of SNPs.

The radiochemical purity for STF particles using ^89^Zr (ox)_2_ was ≥99%. Chelators were not used, and the reaction was carried out at 90 °C for 40 m so that ^89^Zr could be directly labeled on the particle surface. The in-vitro stability of ^89^Zr was assessed in human serum and saline and maintained at ≥90% for 2 h. The effect of ^89^Zr on the surfaces of SNPs was investigated. After 7 days of observation, the sample in saline showed a high stability of 80%, while the stability of the sample in human serum decreased to 60%. In the long-term stability evaluation, ^89^Zr is thought to be lost to some extent due to blood proteins on the surface of STF particles. The labeling stability of ^89^Zr was less than 60%, but it maintained enough intensity for the PET study, and a chelator-free labeling method was selected to prevent changes in the surface properties of nanoparticles caused by chelators.

The cellular uptake of STF-1~5 samples was compared in CT-26 cancer cells. Samples showed the highest cell uptake at 1 h after sample addition, and this gradually decreased over 48 h. Among the five samples, STF-1 and STF-5 showed high uptake, and STF-2 showed the lowest uptake. STF-1 was the most folic-acid-abundant sample and showed high uptake into FR positive CT-26 cells. STF-5 was the most TPP-abundant sample. In vitro, the cell uptake of SFT-1 and STF-5 was similar. In the cell internalization evaluation of STF-1 and STF-5, STF-1 showed the maximum internalization rate over 2 h, and the continuous increase in internalization of STF-5 was confirmed over 24 h. These results are due to differences in the uptake mechanisms of the two samples.

In the PET study using CT-26-bearing mice, both samples accumulated in the liver for 1 h after injection before accumulating in the cancer tissues at 24 h after injection and accumulating in the cancer tissues after 48 h. In STF-5, the intensity observed in the bladder early after injection was due to the clearance through the bladder of TPP separated from STF-5, and this process was observed for over 2 h. Although typical RES accumulation in the liver and spleen was observed, STF-5 showed a higher degree of cancer site accumulation compared to STF-1, thus confirming the difference in drug delivery effect. In addition, it was confirmed that STF-1 retained liver and spleen accumulation for 6 days, whereas STF-5 showed a marked decrease in liver accumulation from day 1 and an increased accumulation in cancer tissue. This is a result of the reported study using the TPP-SNPs; the higher the amount of TPP binding is, the lower the accumulation is in the liver and the faster the removal after accumulation [32].

In this study, combining the TPP on the surface of the drug carrier can be more effective than the FA. This is because, in addition to the results of this study, the TPP can target more diverse types of tumors than the FA, which is dependent on the folate receptor. Nevertheless, it is not always effective to use the TPP alone. This is because TPP with a positive charge is theoretically advantageous for cell internalization but is likely to reach clearance early during blood circulation. Tumor targets using FA are also still effective and need to be properly designed for the purpose of the drug carrier. The overall utility of the two compounds as drug carriers is considered to be an issue that should be discussed continuously.

## 4. Materials and Methods 

### 4.1. Materials

Tetraethyl orthosilicate (TEOS), NH_4_OH, (3-Aminopropyl) triethoxysilane (APTES), triphenylphosphonium bromide (TPP), folic acid (FA), 1-ethyl-3-(3-dimaethylaminopropyl) carbodiimide (EDC), N-hydroxy succinimide (NHS), triethylamine (TEA) and dimethylformamide (DMF) were purchased from Sigma-Aldrich. Dulbecco’s Modified Eagle’s Medium was purchased from Gibco BRL Life Technologies. Carcinoma cells, CT-26 cells were procured from Korean cell line bank (Seoul, Korea) and Balb/c mice (20 ± 1.5 g, female) were purchased from Orient Bio (Seongnam, Korea). ^89^Zr was produced using RFT-30 (30 MeV indigenous cyclotron) at Korea Atomic Energy Research Institute (KAERI). Dynamic light scattering analysis was measured using Zeta-sizer (Malvern, UK). Radioactivity was measured using a CRC-15R ionizing chamber (Capintec, Florham Park, NJ, USA). Radiochemical yield was assessed using an AR-2000 radio-TLC imaging scanner (Bioscan, Santa Barbara, CA, USA). The cell uptake of the radiolabeled SNPs was measured with a Wizard-1470 automatic gamma counter (Perkin Elmer, Waltham, MA, USA). Small animal PET imaging was carried out using a Genesis 4 (Sofie Biosciences, Culver City, CA, USA) machine.

### 4.2. Synthesis of TPP Conjugated SNPs (ST)

SNPs were prepared using TEOS as a silicon source and an added base catalyst (NH_4_OH) and distilled water for hydrolyzing TEOS. The surfaces of synthesized SNPs were then modified with primary amine, 50 mg of SNPs and 2 mL of ethanol were added to a 10 mL flask, and the mixture was stirred at 800 rpm to sufficiently disperse the nanoparticles in the solvent. A total of 0.6 mmol (3-Aminopropyl) triethoxysilane (APTES) was added to the nanoparticle-dispersed solvent, and the mixture was stirred at room temperature for 12 h. After 12 h, the solvent and nanoparticles were separated by centrifugation. Then, nanoparticles were washed two times with distilled water and two times with ethanol to remove unreacted APTES. 

The 1 mg of amine-modified SNPs and 1 mL of DMF were added to five flasks. TPP was added after stirring for 1 min to sufficiently disperse the nanoparticles. The amounts of TPP added to the four flasks varied, and were 0.2, 0.6, 1.8, 3.0, and 4.2 μmol. Then, 1.2 equivalents of EDC, 1.2 equivalents of NHS, and 2.0 equivalents of TEA were added per equivalent of TPP. After stirring at room temperature for 12 h, the nanoparticles were separated from the solvent by centrifugation. To remove unreacted TPP, they were washed 2 times with DMF, 2 times with distilled water, and 2 times with ethanol and then dried at room temperature.

### 4.3. Synthesis of FA Conjugated ST (STF)

One mg of ST and 1 mL of DMSO were added to five 5-mL flasks. A total of 4.2 μmol of FA was added after 1 min to disperse the nanoparticles sufficiently. Then, 1.2 equivalents of EDC, 1.2 equivalents of NHS, and 2.0 equivalents of TEA were added per equivalent of folic acid. After stirring at room temperature for 12 h, the nanoparticles were separated using centrifugation and were washed 2 times with DMSO, distilled water, and ethanol.

### 4.4. Zr-89 Radiolabeling

The five samples were each placed in 5-mL vials, and each 1 mg was dispersed in 100 μL of HEPES. Then, 37 MBq of ^89^Zr (ox)_2_ was added into each vial at 95 °C for 1 h. After completion of the reaction, the solvent and nanoparticles were separated from each other by centrifugation and washed two times with 50 mM DTPA solution and physiological saline to remove free Zr-89. Centrifugation was carried out at 10,000 rpm for 5 min. The ^89^Zr-labeled STFs were dispersed in 100 μL of physiological saline for biological evaluation.

### 4.5. Partition Coefficient (Log P Value)

Ten μL of ^89^Zr-STFs of 37 MBq/mL was added to a test tube containing a mixed solvent (600 μL of PBS as a water-soluble solvent and 600 μL of 1-octanol as a fat-soluble solvent). After vigorous mixing, the mixture was centrifuged at 10,000 rpm for 5 min to separate the two solvents with different densities. The radioactivity of the two solvents was then measured using a gamma ray measuring device.

### 4.6. In-Vitro Stability Studies

One hundred μL of ^89^Zr-STFs (37 MBq/mL) was added to 1 mL of human serum and saline. Tubes were stirred for 7 d at 37 °C. At selected time intervals (15, 30, 60, 120 min and 1 2, and 3 d), the in-vitro stability of the five samples was evaluated using a Radio-TLC scanner. Radio-TLC was measured with i-TLC-SG (1.0 × 10 cm) in the 50 mM DTPA.

### 4.7. Cell Viability Study (MTT Assay)

A cell viability study was carried out using an MTT assay, which indicates the index of mitochondrial dehydrogenase activity. CT-26 cells were aliquoted into 96-well plates at a concentration of 1.0 × 10^5^ cells/well. Twenty-four hours after the dispensation, 100 μL of the medium containing each sample was added and cultured for 48 h. The plate was incubated with MTT solution for 4 h. After forming formazan, 100 μL of dimethyl sulfoxide was added to dissolve the formazan, and the absorbance was measured at 540 nm.

### 4.8. Cellular Uptake

For cellular uptake, 1.0 × 10^5^ CT-26 cells were seeded into 24-well plates and grown in DMEM medium containing 10% (*v*/*v*) fetal bovine serum (FBS) at 37 °C for 24 h. The ^89^Zr-STFs (0.19 MBq/240 μL) was added to CT-26 and incubated for 48 h at 37 °C. The samples were washed two times with PBS, and ^89^Zr-STFs uptake rates were detected using a gamma ray counter at 0.5, 1, 3, 24, and 48 h.

### 4.9. Cell Internalization Assay 

^89^Zr-STF-1 and ^89^Zr-STF-5 were distributed in DMEM containing 10% FBS and added nanoparticles dispersed (0.19 MBq/400 μL) in 24-well plates seeded with CT-26 cells 24 h earlier. After adding nanoparticles, they were cultured in the CO_2_ incubator (37 °C, 5% CO_2_) for 1, 2, 4, 24 h, and the supernatant containing non-internalized particles was collected separately in the tube at each time point. The cells were washed two times with PBS. Afterwards, the cells were treated with 0.1M sodium citrate for 5 min to remove non-internalized particles adsorbed in cell membrane proteins, and cell internalization was measured with a gamma ray counter.

### 4.10. PET Imaging

PET studies were conducted in accordance with the animal experimental guidelines and ethics approved by Korea Atomic Energy Research Institute (IACUC-2015-004). PET images were measured to determine the in-vivo distribution of ^89^Zr-STFs. CT-26 cells were dispersed in saline and injected subcutaneously into the thighs of Balb/c mice (5 × 10^6^ per mouse). After the length of tumors had grown to 120 mm, each mouse was injected intravenously with ^89^Zr-STF-1 and ^89^Zr-STF-5 (3.7 MBq/100 μL per mouse). The mice were anesthetized with isoflurane, and whole-animal imaging was measured using PET for 6 h. We also injected intravenously with ^89^Zr-labeled SNP without chelator and ^89^Zr-oxalate (3.7 MBq/100 μL per mouse) in support of the in-vivo results of STF-1,5.

## 5. Conclusions 

In this study, TPP and FA were bound to SNPs at different ratios, and ^89^Zr was labeled to observe the cell uptake of the samples and PET imaging over time. The samples showed low cytotoxicity and high cell uptake. In the in-vivo experiments conducted on the samples with the highest cell uptake, it was confirmed that STF-5 had the highest TPP ratio accumulation in cancer tissues. In conclusion, although STF-1 and STF-5 had similar cell uptake, the in-vivo drug delivery capacities were different. In-vivo tumor accumulation of SNP confirmed that the target effect by membrane potential difference was more effective than the FR-mediated target effect. In addition, in the preceding study using another proton-emitting nuclide of ^18^F (t_1/2_ = 108 min), the short half-life of ^18^F was limited in the study of long-term in-vivo behavior [32], whereas, in this study using ^89^Zr (t_1/2_ = 3.3 days), it was sufficient to study the in-vivo behavior of nanoparticles for 6 days. It is expected that various in-vivo behavior and tumor targeting studies using ^89^Zr as a tracer will be conducted.

## Figures and Tables

**Table 1:** Labeling yield and radiochemical purity of ^89^Zr-labeled STF-1 to 5 after purification. Stationary phase: i-TLC-SG (1.0 × 10 cm), mobile phase: 50 mM of DTPA.

Samples	Labeling Yield (%)	Radiochemical Purity (%)
STF-1	67.5 ± 28.6	98.7 ± 0.95
STF-2	68.7 ± 3.70	99.6 ± 0.26
STF-3	70.3 ± 2.36	99.8 ± 0.17
STF-4	69.3 ± 1.59	99.6 ± 0.26
STF-5	71.2 ± 2.99	99.8 ± 0.10
Mean	69.4 ± 1.43	99.5 ± 0.46
